# A case of renal cell carcinoma with microscopic pulmonary tumor embolism proven by surgical lung biopsy

**DOI:** 10.1016/j.rmcr.2022.101716

**Published:** 2022-07-31

**Authors:** Takuma Katano, Toyonori Tsuzuki, Hiroki Numanami, Naoto Sassa, Toshio Kato, Akihito Kubo, Satoru Ito

**Affiliations:** a Department of Respiratory Medicine and Allergology, Aichi Medical University, Nagakute, Japan; b Department of Surgical Pathology, Aichi Medical University, Nagakute, Japan; c Division of Chest Surgery, Department of Surgery, Aichi Medical University, Nagakute, Japan; d Department of Urology, Aichi Medical University, Nagakute, Japan; e Medical Oncology, Oncology Center, Aichi Medical University Hospital, Nagakute, Japan

**Keywords:** Pulmonary tumor embolism, Renal cell carcinoma, Respiratory failure, Surgical lung biopsy, Micronodules, GGO, ground glass opacities, mMRC, modified Medical Research Council dyspnea scale, PTE, pulmonary tumor embolism, SpO_2_, peripheral oxygen saturation

## Abstract

Pulmonary tumor embolism (PTE) is difficult to diagnose before death. We report the case of a 75-year-old man with microscopic PTE of renal cell carcinoma who was diagnosed by surgical lung biopsy. He visited our hospital because of dyspnea on exertion. Chest computed tomography (CT) showed multiple micronodules and ground glass opacities. Steroid therapy was started as therapeutic diagnosis for IgG4-related pulmonary disease. However, he was admitted our hospital due to progressive respiratory failure. Pathological findings of a lung biopsy obtained by video-assisted thoracic surgery showed PTE of renal cell carcinoma without embolization of large pulmonary arteries. He received palliative medicine and died four months after the surgical lung biopsy. In cases of multiple micronodules in chest CT findings and worsened respiratory symptoms, PTE should be considered in the differential diagnosis.

## 1 Introduction

Pulmonary tumor embolism (PTE), first described by Schmidt in 1897 [1], is one of the causes of respiratory failure and mortality in patients with malignant tumors such as carcinomas of breast, stomach, liver, and kidney [[2], [3], [4], [5], [6]]. PTE may appear as the first presentation of a malignancy [6,7]. Pathological characteristics of PTE involve the presence of isolated tumor cells or clusters of tumor cells within the pulmonary arterial system from large pulmonary arteries to alveolar septal capillaries [3,8]. Massive tumor embolism of renal cell carcinoma in the proximal pulmonary artery has been reported [5,6,9]. However, PTE, specifically a microscopic one, is difficult to detect before death [10]. Here, we describe a case of unsuspected microscopic PTE of renal cell carcinoma, diagnosed by surgical lung biopsy, whose chest computed tomography (CT) showed multiple micronodules and ground grass opacities (GGOs).

## 2 Case presentation

**Fig. 1:**
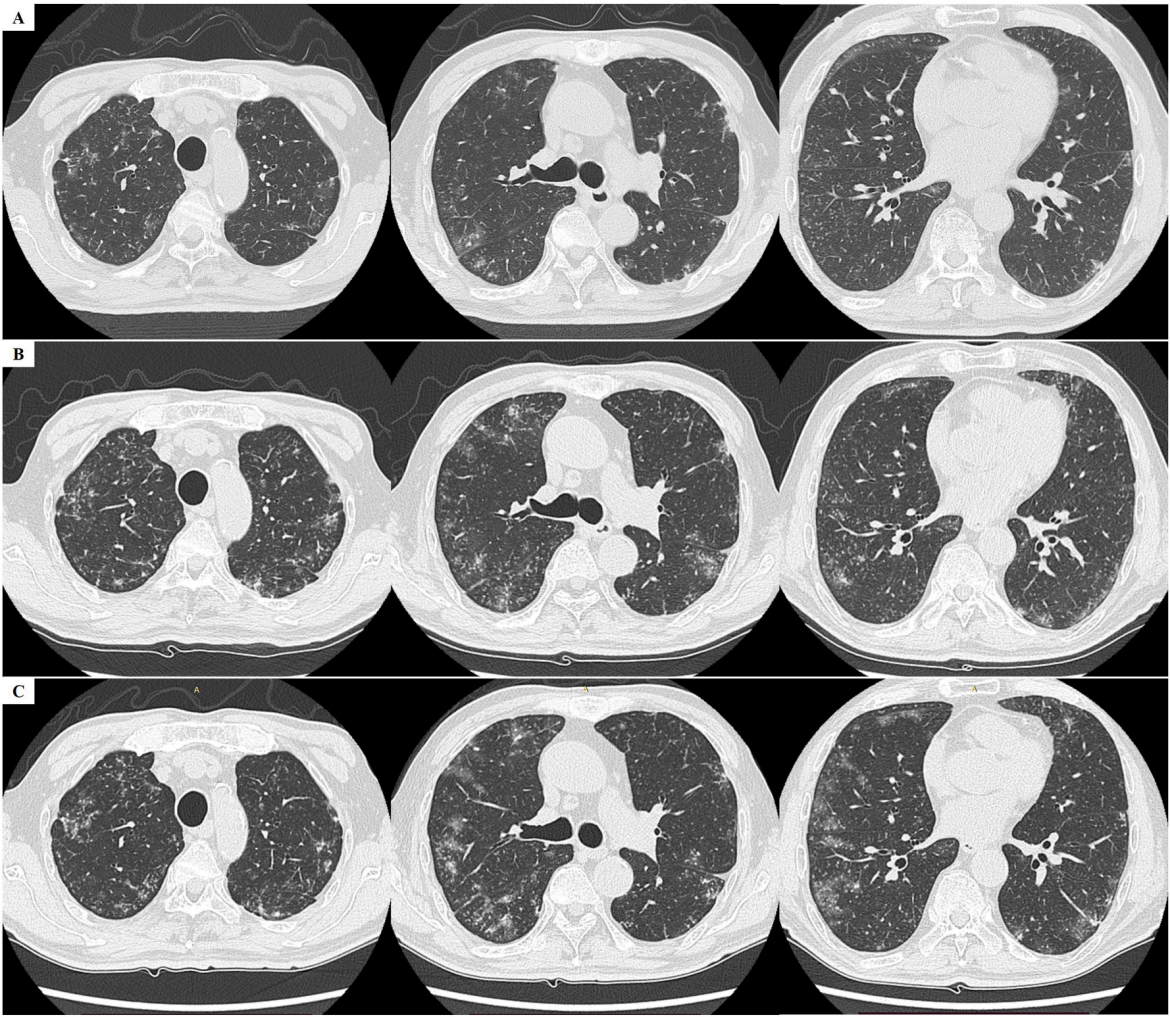
Images of chest computed tomography (CT). Images of chest CT at the initial visit (A), at the admission (B), and before the surgical lung biopsy (C). Expansion of the area of multiple micronodules with surrounding ground glass opacities in both lungs were shown.

A 75-year-old man visited the Department of General Medicine, Aichi Medical University Hospital, with a complaint of dyspnea on exertion of the modified Medical Research Council dyspnea scale (mMRC) 1. He had hypertension and type 2 diabetes mellitus but no previous history of smoking or respiratory diseases. Because his chest CT showed multiple micronodules with surrounding GGOs in both lungs (Fig. 1A) and slight enlargement of mediastinal lymph nodes, he was referred to the Department of Respiratory Medicine and Allergology. Peripheral oxygen saturation (SpO_2_) was 98%. There was no microscopic hematuria. Serum tumor markers, carcinoembryonic antigen (0.8 ng/mL), squamous cell carcinoma antigen (1.8 ng/mL), and CYFRA (2.1 ng/mL), were not elevated. The levels of serum IgG (2284 mg/dL) and IgG4 (779 mg/dL) were high. The results of a sputum smear for acid-fast bacilli, sputum PCR for *Mycobacterium tuberculosis*, and serum interferon gamma release assay were all negative. His pulmonary function, including vital capacity (101.6% of the predicted value), forced vital capacity (105.1%), forced expiratory volume in 1 s (124.9%), and diffusing capacity of the lungs for carbon monoxide (112.6%), were preserved. The following diagnostic possibilities were considered: pulmonary infection including tuberculosis and mycosis, interstitial lung disease, and malignancy.

Two weeks after his first visit, bronchoalveolar lavage (BAL) and *trans*-bronchial needle aspiration from a mediastinal lymph node were performed using a flexible bronchoscope. The results of BAL fluid obtained from the B^5^ right middle lobe showed elevation of lymphocytes (13%) and eosinophils (10%). However, a definitive diagnosis could not be made by bronchoscopy results. A pulmonary manifestation of IgG4-related disease was suspected, and prednisolone 20 mg per day was initiated two weeks after the bronchoscopy. However, the steroid treatment was not effective. He was admitted to the Department of Respiratory Medicine and Allergology due to worsening dyspnea to mMRC 4. On admission, his SpO_2_ level had dropped to 90% in room air. Transthoracic echocardiography showed a normal left ventricular ejection fraction (70%) and a tricuspid regurgitation pressure gradient of 11 mmHg with no evidence of pulmonary hypertension. His chest CT showed increases in the areas of micronodules and GGOs (Fig. 1B) compared with those at the initial visit (Fig. 1A).

**Fig. 2:**
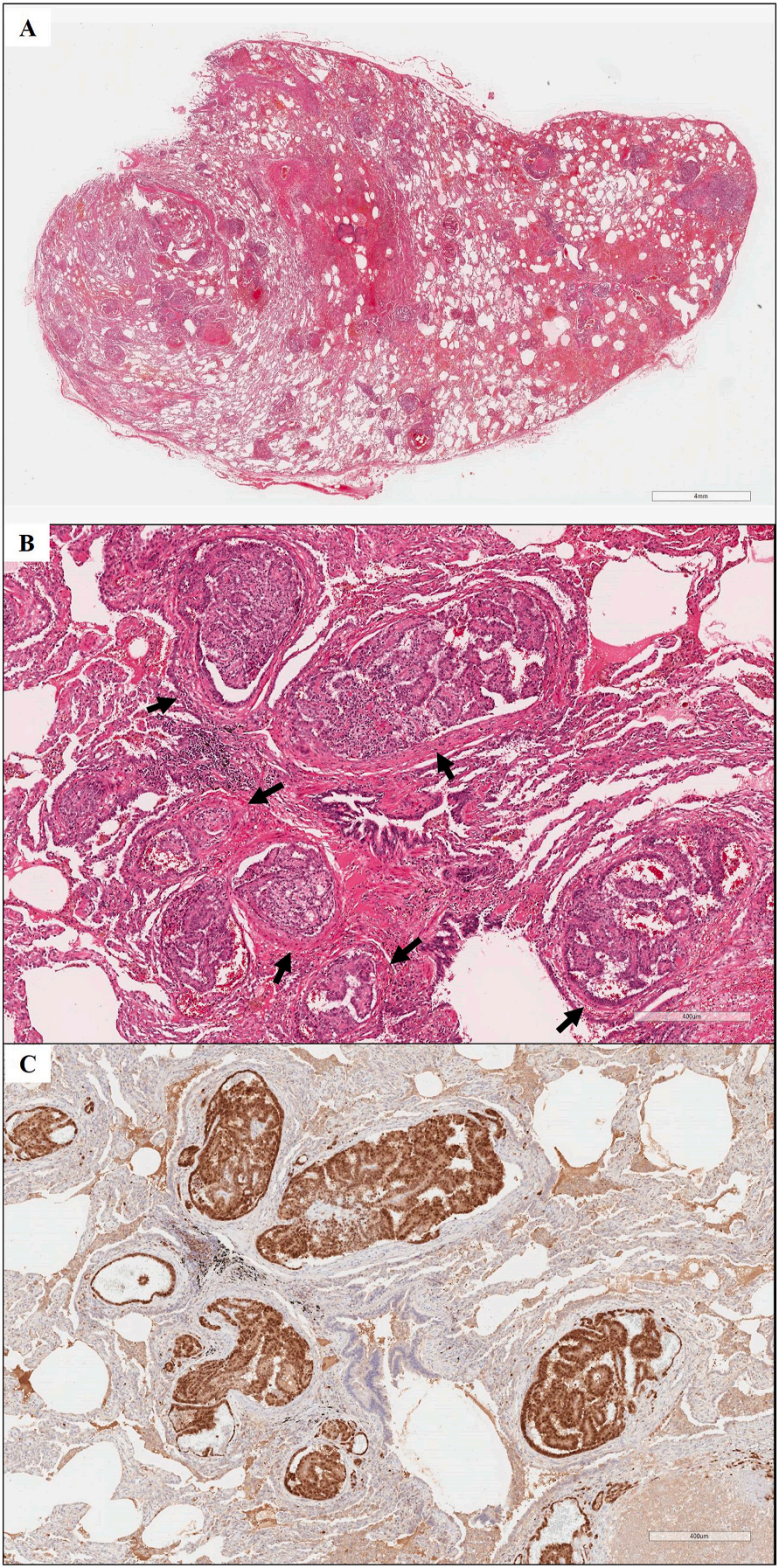
Histopathological findings of lung specimens obtained by surgical lung biopsy. (A) A low power view of the lung specimen showed tumor embolization in nearly all small pulmonary arterioles associated with dilation (hematoxylin-eosin). (B) A high-power view (hematoxylin-eosin) showed tumor cells formed by renal cell carcinoma cells with papillary or glandular structures in. The arrows indicate the muscle layer of the arteries. (C) Immunostaining for PAX8 showed tumor cells positivity for PAX8.

**Fig. 3:**
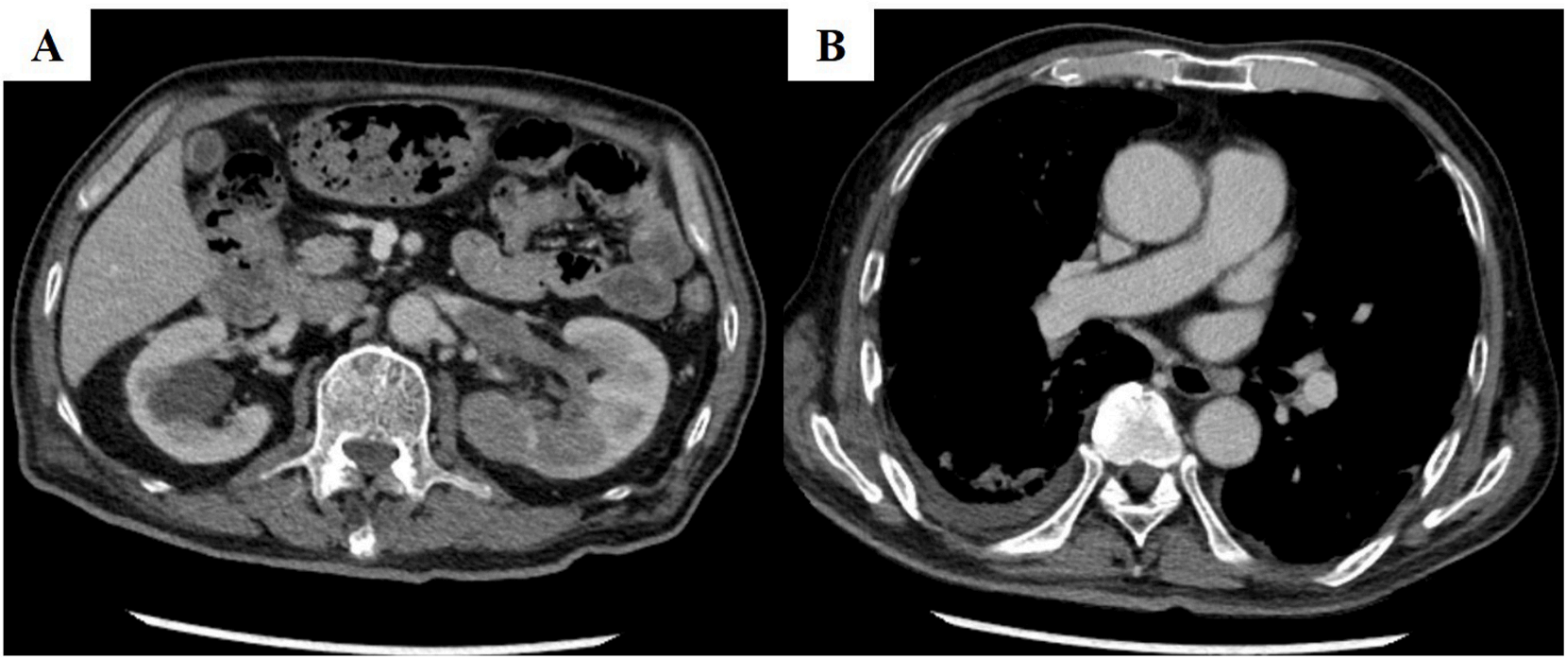
Images of enhanced-contrast CT findings at diagnosis of renal cell carcinoma. (A) An internal heterogeneous mass in the morphologically preserved left kidney and invasion of the left renal vein are shown. (B) There were no massive tumor emboli in large pulmonary arteries.

After admission, the dose of prednisolone was increased to 50 mg per day, but partial pressure of oxygen in arterial blood dropped to 55.4 mmHg with 1 L/min supplemental oxygen via nasal cannula and further radiological deterioration was confirmed on chest CT (Fig. 1C). Surgical lung biopsy for confirming the histopathological diagnosis was considered but challenging because of his age and respiratory failure. After informed consent was obtained from the patient and his family, surgical lung biopsy of the right middle lobe of the lung using a video-assisted thoracoscope for the purpose of differential diagnosis was performed two weeks after the admission. The lung biopsy revealed embolization of tumor cells into nearly all small pulmonary arterioles associated with extensive dilation. (Fig. 2A and B). Renal cell carcinoma was morphologically suspected. The tumor cells were positive for PAX8 (Fig. 2C) but negative for thyroid transcription factor-1, which finding supported renal cell carcinoma origin. A whole-body contrast-enhanced CT to identify the primary lesion showed a heterogeneous mass invading the left renal vein in the left kidney (Fig. 3A), but no massive embolus in the main pulmonary arteries (Fig. 3B). He was finally diagnosed with renal cell carcinoma of clinical T3aN0M1 stage IV. He elected not to receive systemic chemotherapy but to receive palliative care and supplemental oxygen therapy. One month after the surgery, he received a transfusion of red blood cells for severe anemia due to appearance of gross hematuria. He died four months after the surgical lung biopsy at another hospital for hospice care.

## 3 Discussion

This case initially presented with dyspnea on exertion, multiple micronodules and surrounding GGOs on chest CT, and a high level of serum IgG4. Unexpectedly, the histopathology of a surgical lung biopsy demonstrated characteristics of PTE of renal cell carcinoma. To our knowledge, this the first report of microscopic PTE of renal cell carcinoma diagnosed by a surgical lung biopsy.

Interestingly, chest CT images at the initial visit and before surgery showed micronodules in a tree-in-bud pattern and GGOs in both lungs (Fig. 1). The tree-in-bud pattern generally reflects bronchiolar lesions such as pulmonary tuberculosis, aspiration pneumonia, and IgG4-related lung disease [11,12]. However, it has been reported that the tree-in-bud pattern in CT reflects histopathological characteristics of PTE, such as filling of centrilobular arteries with tumor cells and fibrocellular intimal hyperplasia in small arteries and arterioles [13,14]. In our present case, micronodules on the CT reflected pathologically innumerable tumor emboli. The appearance of GGOs is consistent with the findings of Yoshii et al. [15].

Contrast-enhanced CT after the surgical lung biopsy showed involvement of the renal veins but no massive tumor emboli to proximal vessels such as the main pulmonary artery (Fig. 3B). In light of the CT findings, the histopathology of the surgical lung biopsy specimen revealed microscopic tumor emboli within small pulmonary arterioles with invasion of alveoli by carcinoma cells. Our results are consistent with findings in a previous report that tumor emboli are caused when the primary tumor invades the renal vein in renal cell carcinoma [2]. Kane et al. divided PTE into the following four types: 1) large, proximal emboli, 2) generalized lymphatic involvement, 3) pure microscopic tumor emboli involving the small arteries or arterioles, and 4) some combination of these [16]. Of these four types, distinguishing between the clinical presentation of lymphatic and microvascular disease can be challenging [10], but Soares reported that they are morphologically different entities and respiratory distress is more likely to be the main cause of death in PTE [17]. Based on histopathological and radiological findings, the present case was the third microscopic type, which gradually spread from renal vein lesions.

The diagnosis of renal carcinoma was made by the surgical lung biopsy and subsequent contrast-enhanced CT. There was no gross or microscopic hematuria at his first visit, and the initial plane CT did not show any obvious mass in the left kidney. It is known that massive proximal emboli cause rapid onset of acute right heart failure and progressive cor pulmonale [10]. However, our patient did not show any evidence of pulmonary hypertension or cor pulmonale despite progressive respiratory failure with normal coagulation results. The lack of hematuria, cor pulmonale, or an abnormal coagulation system led us not to suspect PTE or renal cell carcinoma. In addition, our present case showed high serum IgG4 levels. Su et al. reported that high serum IgG4 levels were observed in patients with malignant tumors without IgG4-related diseases [18]. However, a role of IgG4 in pathophysiology of malignant tumors is unclear.

In conclusion, in cases of micronodules and surrounding GGOs in chest CT findings and advanced respiratory symptoms, it is important to consider possibilities of PTE of malignant tumors including renal cell carcinoma.

## Funding

This research did not receive any specific grant from funding agencies in the public, commercial, or not-for-profit sectors.

## Authorship statement

T.Katano and SI were responsible for the present study's design, as well as for data acquisition, analysis, interpretation, and drafting of the manuscript. T.Kato and HN collected the data. TT was responsible for the histopathological analysis. AK and MS supervised the research work and manuscript. SI was responsible for data acquisition, and drafting of the manuscript. All the authors have approved the submission of the manuscript and meet the ICMJE authorship criteria.

## Declaration of competing interest

The authors have no conflicts of interest to declare.

## References

[1] Schmidt M.B. (1897). Ueber Krebszellenemboliem in dem Lungenarterien. Zentralbl. Allg. Pathol..

[2] Winterbauer R.H., Elfenbein I.B., Ball W.C. (1968). Incidence and clinical significance of tumor embolization to the lungs. Am. J. Med..

[3] Bassiri A.G., Haghighi B., Doyle R.L., Berry G.J., Rizk N.W. (1997). Pulmonary tumor embolism. Am. J. Respir. Crit. Care Med..

[4] Agrawal A., Sahni S., Iftikhar A., Talwar A. (2015). Pulmonary manifestations of renal cell carcinoma. Respir. Med..

[5] Kubota H., Furuse A., Kotsuka Y., Yagyu K., Kawauchi M., Saito H. (1996). Successful management of massive pulmonary tumor embolism from renal cell carcinoma. Ann. Thorac. Surg..

[6] Shim H., Kim W.S., Kim Y.W., Yang S.S., Kim D.K. (2012). Successful management of pulmonary and inferior vena cava tumor embolism from renal cell carcinoma. Kor. J. Thorac. Cardiovasc. Surg..

[7] Veinot J.P., Ford S.E., Price R.G. (1992). Subacute cor pulmonale due to tumor embolization. Arch. Pathol. Lab Med..

[8] Soares F.A., Landell G.A., de Oliveira J.A. (1992). Pulmonary tumor embolism to alveolar septal capillaries. An unusual cause of sudden cor pulmonale. Arch. Pathol. Lab Med..

[9] Wieder J.A., Laks H., Freitas D., Marmureanu A., Belldegrun A. (2003). Renal cell carcinoma with tumor thrombus extension into the proximal pulmonary artery. J. Urol..

[10] Roberts K.E., Hamele-Bena D., Saqi A. (2003). Pulmonary tumor embolism: a review of the literature. Am. J. Med..

[11] Collins J., Blankenbaker D., Stern E.J. (1998). CT patterns of bronchiolar disease: what is “tree-in-bud”. AJR Am. J. Roentgenol..

[12] Chen C.F., Chu K.A., Tseng Y.C., Wu C.C., Lai R.S. (2017). IgG4-related lung disease presenting as interstitial lung disease with bronchiolitis. A case report. Medicine (Baltim.).

[13] Tack D., Nollevaux M.C., Gevenois P.A. (2001). Tree-in-bud pattern in neoplastic pulmonary emboli. AJR Am. J. Roentgenol..

[14] Franquet T., Giménez A., Prats R., Rodríguez-Arias J.M., Rodríguez C. (2002). Thrombotic microangiopathy of pulmonary tumors: a vascular cause of tree-in-bud pattern on CT. AJR Am. J. Roentgenol..

[15] Yoshii Y., Kawabata Y., Takayanagi N., Araya J., Kuwano K., Sugita Y. (2015). Progressive diffuse pulmonary interstitial opacities due to complications of pulmonary tumor emboli: an autopsy case report. Intern. Med..

[16] Kane R.D., Hawkins H.K., Miller J.A., Noce P.S. (1975). Pulmonary tumor emboli. Cancer.

[17] Soares F.A., Pinto A.P., Landell G.A., de Oliveira J.A. (1993). Pulmonary tumor embolism to arterial vessels and carcinomatous lymphangitis. A comparative clinicopathological study. Arch. Pathol. Lab Med..

[18] Su Y., Sun W., Wang C., Wu X., Miao Y., Xiong H., Bai L., Dong L. (2015). Detection of serum IgG4 levels in patients with IgG4-related disease and other disorders. PLoS One.

